# Further Botanical Exploration of Limestone Karst Habitats in Northeastern Thailand Reveals a New Yellow-Flowered Species of *Boesenbergia* Kuntze (Zingiberaceae) †

**DOI:** 10.3390/life16091480

**Published:** 2026-09-04

**Authors:** Thawatphong Boonma, Surapon Saensouk, Piyaporn Saensouk, Mukdamas Ruengrit, Pathomthat Srisuk, Prasert Ruannakarn, Min Khant Naing

**Affiliations:** 1 Diversity of Family Zingiberaceae and Vascular Plant for Its Applications Research Unit, Walai Rukhavej Botanical Research Institute, Mahasarakham University, Maha Sarakham 44150, Thailand; boonma.thawat@gmail.com; 2 Diversity of Family Zingiberaceae and Vascular Plant for Its Applications Research Unit, Department of Biology, Faculty of Science, Mahasarakham University, Maha Sarakham 44150, Thailand; 3 Independent Researcher, Khon Kaen 40350, Thailand; mukdamas0900@gmail.com; 4 Department of Pharmaceutical Technology, Faculty of Pharmaceutical Sciences, Khon Kaen University, Khon Kaen 40002, Thailand; spatho@kku.ac.th; 5 Department of Educational Research and Development, Faculty of Education, Mahasarakham University, Maha Sarakham 44000, Thailand; prasert.rua@msu.ac.th; 6 Biodiversity Research Center, Hpa An 13011, Myanmar; minkhantnaingminkhantnaing44@gmail.com

**Keywords:** *Boesenbergia*, karst, limestone, new species, northeastern, taxonomy, Thailand

## Abstract

Limestone karst habitats in tropical Asia support specialized and geographically restricted plant diversity, yet many areas remain incompletely explored. During botanical surveys of limestone habitats in northeastern Thailand, a distinctive, yellow-flowered population of *Boesenbergia* was discovered in Phu Pha Man District, Khon Kaen Province. Morphological observations were conducted on living plants in the natural population and spirit-preserved material, supplemented by comparisons with protologues, published descriptions, and herbarium material. Patterns of phenetic similarity among ten selected *Boesenbergia* taxa were evaluated using 32 morphological characters, Gower distance, UPGMA clustering, and principal coordinates analysis (PCoA). The population is described here as *Boesenbergia calcicola* Boonma, Saensouk & P.Saensouk, sp. nov. It is morphologically most comparable to *B. petiolata* but differs by its conspicuously exserted inflorescence, shorter bracts and flowers, distally puberulent floral tube, smaller puberulent lateral staminodes, and distinctive labellum color pattern. Phenetic analyses were used to summarize patterns of overall morphological similarity among the examined taxa. The species is currently known only from its limestone type locality at approximately 385 m a.s.l. and is provisionally assessed as Data Deficient (DD). This discovery further documents localized *Boesenbergia* diversity associated with limestone karst habitats in northeastern Thailand.

## 1. Introduction

Limestone karst landscapes are among the most environmentally heterogeneous and spatially fragmented ecosystems in tropical Asia [1,2]. Shallow alkaline soils, exposed rock surfaces, fissures, shaded cliffs, and fine-scale variation in moisture create specialized microhabitats that often support plants with narrow ecological requirements and restricted distributions [1,2,3]. Individual limestone outcrops often harbor site-restricted or hyper-endemic taxa, while their small and highly localized populations are particularly vulnerable to habitat disturbance and environmental change [1,4,5]. Nevertheless, many limestone areas remain insufficiently explored, particularly for seasonal herbs that emerge and flower during a brief period once each year. Repeated surveys during appropriate flowering periods are therefore essential for documenting their diversity and detecting geographically restricted taxa.

*Boesenbergia* Kuntze is a predominantly tropical Asian genus of perennial rhizomatous herbs, many of which occur in seasonal forest understories and specialized rocky habitats [5,6,7]. The circumscription of the genus has been revised following morphological and molecular studies, but the limits of several species remain difficult to interpret because vegetative characters frequently overlap and diagnostically important floral structures may be distorted or obscured in preserved specimens [7,8]. Species delimitation therefore continues to depend heavily on observations of living flowering plants, supported by comparisons with type material, protologues, herbarium specimens, and regional taxonomic treatments [9,10]. This is particularly important among yellow-flowered species, in which similar overall coloration may conceal substantial differences in inflorescence architecture, floral indumentum, labellum configuration, lateral staminodes, and androecial structures. Among the currently known species, *B. petiolata* Sirirugsa [11] and *B. moodii* Nob. Tanaka & Paing [7,12,13] provide the most relevant morphological comparisons for the material examined here.

The recently described species, *Boesenbergia lamudiae* Boonma, Saensouk & P.Saensouk [5] from a localized limestone habitat in Loei Province demonstrated that the karst landscapes of northeastern Thailand continue to harbor previously undocumented and narrowly distributed species. Subsequent botanical exploration of limestone habitats in Khon Kaen Province revealed another distinctive population of *Boesenbergia*. The plants are currently known only from a limestone-associated habitat and are readily recognized in the field by their terminal inflorescences prominently exserted beyond the leafy shoots, distally puberulent floral tubes, consistently puberulent lateral staminodes, and yellow labella bearing a distinctive deep-red median band with paired dark-maroon basal patches. These characters are not encompassed by the documented intraspecific variation of *B. petiolata* [7,11,12,13,14,15], which primarily concerns leaf robustness, lamina-base shape, abaxial pigmentation, and floral coloration.

The present study describes and illustrates this taxon as a new species, compares it with *B. petiolata*, *B. moodii*, and other morphologically relevant congeners, and documents its habitat, phenology, distribution, and provisional conservation status. Morphological differentiation was further evaluated using Gower distance, UPGMA clustering, and principal coordinates analysis [16,17]. Recognition of the taxon as a distinct species is based on a consistent combination of diagnostic morphological characters, whereas Gower distance, UPGMA clustering, and PCoA are used only to summarize overall morphological similarity among the examined taxa. The discovery further emphasizes the importance of continued botanical exploration in the incompletely surveyed limestone landscapes of northeastern Thailand, where additional narrowly distributed and potentially endemic members of Zingiberaceae may remain undocumented.

## 2. Materials and Methods

### 2.1. Field Observations and Study Material

**Figure 1:**
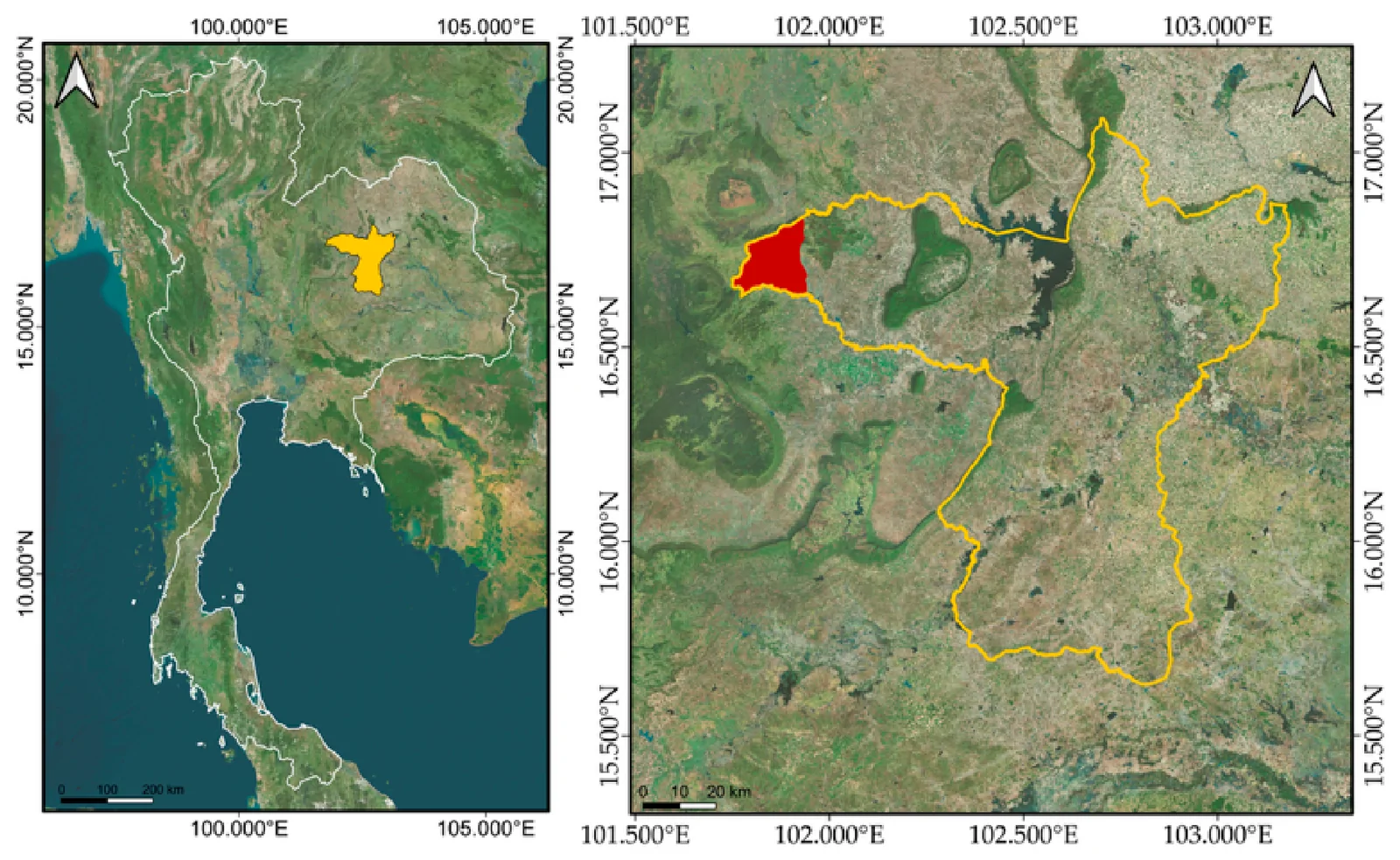
Location of the study area of *Boesenbergia calcicola* Boonma, Saensouk & P.Saensouk, sp. nov. in Phu Pha Man District, Khon Kaen Province, northeastern Thailand. The left map shows the location of Khon Kaen Province (yellow) within Thailand. The right map shows Khon Kaen Province outlined in yellow, with Phu Pha Man District, the type locality of *B. calcicola*, highlighted in red. Map prepared by Kamonwan Koompoot.

The new species was studied from living flowering plants occurring in a limestone-associated habitat in Phu Pha Man District, Khon Kaen Province, northeastern Thailand (Figure 1). The precise collection coordinates are intentionally withheld from publication to reduce potential collection pressure on the currently known population; consequently, the locality is presented at the district level in Figure 1. Field observations were conducted during two consecutive flowering seasons, on 31 July 2025 and 13 July 2026, to document the plants in their natural habitat and to assess the consistency of vegetative and floral characters among individuals and between seasons. The distribution map was prepared using QGIS version 3.34 with the WGS 84 geographic coordinate reference system (EPSG:4326).

Measurements of habit, underground organs, stems, leaves, and inflorescences were obtained directly from plants examined in their natural habitat. Representative flowers and other delicate reproductive structures were collected from the natural population and preserved in 70% ethanol for subsequent examination and measurement. Floral dimensions and fine morphological characters were therefore assessed using both freshly collected material and spirit-preserved material originating from the same natural population.

Living plants were photographed before collection, and colors were recorded descriptively by direct visual observation of fresh material under natural daylight. No standardized color chart was used. Particular attention was given to rhizome architecture, inflorescence position and degree of exsertion, bract morphology and color, floral-tube indumentum, labellum configuration and color pattern, lateral-staminode morphology and indumentum, and androecial structures. Representative voucher specimens were prepared using standard herbarium techniques and deposited at the Vascular Plant Herbarium, Mahasarakham University (VMSU).

### 2.2. Herbarium Study and Comparative Taxonomic Sources

Herbarium specimens were studied through a combination of direct examination and digital resources. Accessible collections were examined in person, whereas additional material was studied using high-resolution images available through online repositories, including the Chinese Virtual Herbarium [18]. Specimens examined directly were from the Botanic Garden and Botanical Museum Berlin-Dahlem, Germany (B), Bangkok Herbarium, Department of Agriculture, Thailand (BK), Department of National Parks, Wildlife and Plant Conservation, Thailand (BKF), The Natural History Museum, London, England, United Kingdom (BM), University of Copenhagen, Copenhagen, Denmark (C), Botanical Survey of India, Howrah, West Bengal, India (CAL), Medicinal Plants Research Institute, Thailand (DMSC), Royal Botanic Garden Edinburgh, Edinburgh, United Kingdom (E), Vietnam Academy of Science and Technology (VAST), Hanoi, Vietnam (HN), National Institute of Medicinal Materials, Vietnam (HNPM), VNU University of Science, Hanoi, Vietnam (HNU), Guangxi Institute of Botany, China (IBK), Chinese Academy of Medical Sciences, China (IMDY), Khon Kaen University Herbarium, Thailand (KKU), Naturalis Biodiversity Center, Leiden, Netherlands (L), Muséum National d’Histoire Naturelle, Paris, France (P), Queen Sirikit Botanic Garden, The Botanical Garden Organization, Thailand (QBG), Southern Institute of Ecology, Ho Chi Minh City, Vietnam (SGN), Singapore Botanic Gardens, National Parks Board, Singapore (SING), Vascular Plant Herbarium, Mahasarakham University, Thailand (VMSU), Institute of Life Science, Ho Chi Minh City, Vietnam (VNM), and the Vietnam National Museum of Nature, Hanoi, Vietnam (VNMN). Specimens examined exclusively from digital images were from Harvard University, Cambridge, Massachusetts, U.S.A. (A), Aarhus University, Denmark (AAU), Universiti Malaysia Sabah, Kota Kinabalu, Sabah, Malaysia (BORH), Xishuangbanna Tropical Botanical Garden, Academia Sinica, Yunnan, China (HITBC), Royal Botanic Gardens, United Kingdom (K), The New York Botanical Garden, New York, U.S.A. (NY), Forest Research Institute (RAF), National Museum of Nature and Science, Tokyo, Japan (TNS), and the Smithsonian Institution, Washington, Columbia, U.S.A. (US). Herbarium acronyms follow the Index Herbariorum [19].

Type material, protologues, original taxonomic descriptions, regional taxonomic treatments, and available high-resolution images were consulted for the comparative assessment. For the comparative taxa included in the phenetic analysis, protologues and original taxonomic descriptions were used as the primary sources of quantitative morphological data. Available type specimens and high-resolution images of type material were additionally examined to verify diagnostic qualitative characters and character states. Comparative assessments focused on morphologically relevant species of *Boesenbergia*, particularly taxa with terminal inflorescences, predominantly yellow flowers, or floral structures resembling those of the Khon Kaen population. The principal comparisons included *B. petiolata* Sirirugsa and *B. moodii* Nob. Tanaka & Paing, together with *B. bella*, *B. phengklaii*, *B. putiana*, *B. tenuispicata*, *B. truncata*, *B. xiphostachya*, and *B. sugudensis* [7,11,12,13,14,15,20].

Morphological data for *B. petiolata* were primarily based on the most recent treatment in the Flora of Thailand [7], while the original taxonomic treatment of Sirirugsa [11,12] was additionally consulted to verify the historical circumscription and diagnostic morphology of the species.

Published evidence concerning population-level variation within *B. petiolata* was considered when evaluating the diagnostic value of individual characters [15]. Features documented as variable within that species, including leaf robustness, lamina-base shape, abaxial pigmentation, the degree of yellow tinting in the flowers, the intensity of red coloration in the center of the labellum, and violet coloration at the labellum apex, were not treated as primary evidence for species delimitation [15].

### 2.3. Morphological Measurements and Terminology

Morphological measurements were taken from plants examined in their natural habitat and from spirit-preserved material collected directly from the natural population. Larger vegetative and inflorescence structures were measured using a standard ruler and a digital Vernier caliper (Mitutoyo Corporation, Kawasaki, Japan). Floral structures and fine morphological features, including surface indumentum, calyx morphology, labellum configuration, lateral staminodes, anther, stigma, epigynous glands, and ovary, were examined using a stereomicroscope (Stemi 2000-C; Carl Zeiss, Oberkochen, Germany).

Colors were assessed descriptively by direct visual observation of fresh living material under natural daylight. No standardized color chart was used. Morphological terminology follows Beentje [21]. Measurements are presented as ranges, with exceptional values placed in parentheses. Scientific names and nomenclatural information were verified using Plants of the World Online [22] and the International Plant Names Index [23].

### 2.4. Morphological Character Selection and Coding

Morphological characters used in the phenetic analyses were organized into four categories according to the organs from which they were derived: underground organs (UG), vegetative characters (VG), inflorescence characters (IN), and floral characters (FL). Each character was assigned a unique alphanumeric code to ensure consistent scoring and facilitate reference between the character definitions and the coded matrix.

Characters were selected according to their taxonomic informativeness, comparability, and availability across all operational taxonomic units. Ecological, geographical, phenological, and conservation-related variables were excluded so that the analyses represented morphological similarity alone. Characters were also excluded when they were inconsistently documented, potentially strongly influenced by developmental or environmental conditions, based on incompatible measurement conventions, or unavailable for one or more of the included taxa.

The final dataset comprised 32 morphological characters scored across ten operational taxonomic units: *B. calcicola*, *B. petiolata*, *B. bella*, *B. phengklaii*, *B. putiana*, *B. moodii*, *B. tenuispicata*, *B. xiphostachya*, *B. truncata*, and *B. sugudensis*. For the comparative taxa, quantitative and qualitative morphological data were compiled from protologues, original taxonomic descriptions, the Flora of Thailand [7], and recent taxonomic treatments, as appropriate for each taxon. Where quantitative measurements were reported as ranges, the maximum documented values used in the comparative dataset were scored consistently. Qualitative character states were derived from the relevant taxonomic descriptions and verified against available type material or high-resolution images of type specimens whenever possible.

Data types were assigned according to the coding system implemented in PAST. Direct numerical measurements were retained as the default continuous or unspecified data type (“–“); ordered discrete characters were designated as Ordinal; unordered multistate qualitative characters as Nominal; and two-state characters as Binary. The character definitions and coding scheme are provided in Table A1, and the complete morphological character matrix is presented in Table A2.

### 2.5. Phenetic Analyses

Phenetic analyses were conducted using PAST version 4.15 [24]. Pairwise morphological distances among the ten operational taxonomic units were calculated using Gower distance, which accommodates datasets containing both quantitative and qualitative variables. The resulting Gower distance matrix was analyzed using the unweighted pair-group method with arithmetic mean (UPGMA) to summarize overall morphological similarity among the examined taxa. Principal coordinates analysis (PCoA) was performed using the same Gower distance matrix to visualize the relative positions of the taxa in multidimensional morphological space. The percentages represented by the first two principal coordinate axes were obtained directly from PAST. The resulting UPGMA dendrogram and PCoA ordination were interpreted only as phenetic summaries of the scored morphology and not as estimates of phylogenetic relationships, evolutionary independence, or reproductive isolation. The complete Gower distance matrix used for both analyses is provided in Table A3. *Boesenbergia regalis* Kharuk & Tohdam [25] was considered in the qualitative taxonomic assessment but was excluded from the phenetic analyses because several diagnostically important floral structures remain insufficiently documented for complete and consistent scoring. The available treatment was based on photographs and herbarium specimens, while neither the holotype nor verified living material was available for detailed examination. The character scores of the morphologically closest taxa, particularly *B. petiolata*, *B. phengklaii*, *B. putiana*, and *B. xiphostachya*, were rechecked against their protologues, original descriptions, and available type material (Table A4) before the final analyses were performed.

### 2.6. Preliminary Conservation Assessment

The preliminary conservation status of the new species was evaluated following the IUCN Red List Categories and Criteria [26]. The assessment considered its currently known distribution, available population information, habitat specificity, and available information on potential threats. The species is presently known only from the type locality, and adjacent limestone habitats remain incompletely surveyed; the assessment is therefore considered provisional.

### 2.7. Figure Preparation

Photographic plates and figure compositions were prepared using Pixelmator Pro version 3.6.5 (“Archipelago”; Pixelmator Team, Vilnius, Lithuania) on a MacBook Pro (13-inch, M1, 2020; Apple Inc., Cupertino, CA, USA). Scientific illustrations were digitally prepared using an iPad Air (5th generation; Apple Inc.) running iPadOS 17.5.1. All figures were prepared by the first author (T.B.).

## 3. Results

Morphological examination of living plants and spirit-preserved floral material from the Khon Kaen population revealed a consistent combination of vegetative, inflorescence, and floral characters distinguishing the studied plants from morphologically similar species of *Boesenbergia*. The principal diagnostic characters include the terminal inflorescence conspicuously exserted beyond the leafy shoot, relatively short bracts and flowers, a distally puberulent floral tube, small puberulent lateral staminodes, and labellum with a distinctive deep-red median band and dark-maroon basal markings. Comparison with relevant protologues, published descriptions, and herbarium material, particularly those of *B. petiolata*, supported the recognition of the Khon Kaen population as a distinct species, described below as *B. calcicola* Boonma, Saensouk & P.Saensouk sp. nov.

### 3.1. Taxonomic Treatment of New Species

***Boesenbergia calcicola*** **Boonma, Saensouk & P.Saensouk, sp. nov.**

**Type:** THAILAND, Northeastern, Khon Kaen Province, Phu Pha Man District, c. 385 m elevation, 31 July 2025, T. Boonma 2571 (VMSU).

**Figure 2:**
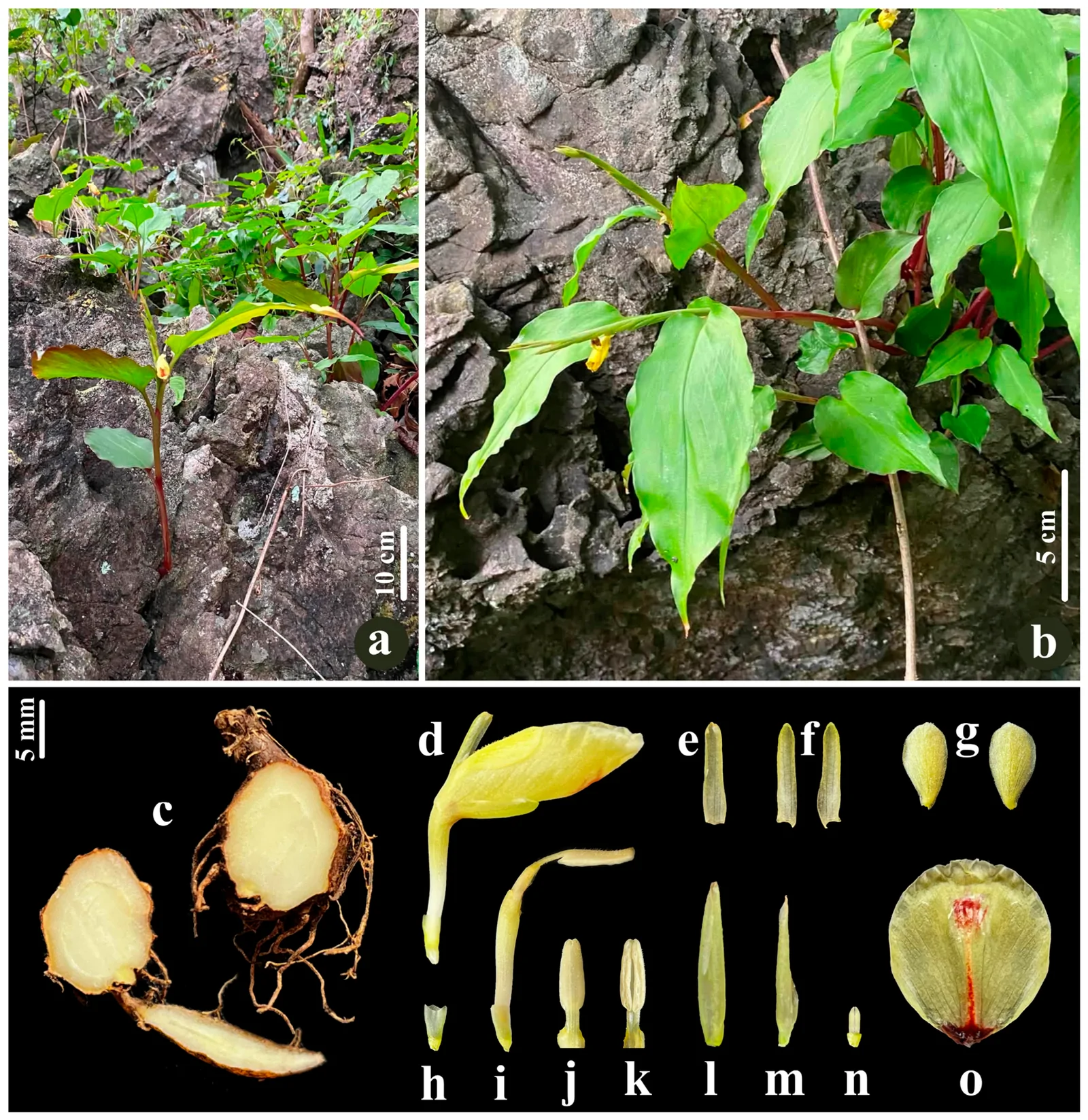
*Boesenbergia calcicola* Boonma, Saensouk & P.Saensouk, sp. nov. (**a**,**b**) Habit in natural habitat. (**c**) Dissected rhizome and tuberous root. (**d**) Lateral view of flower. (**e**) Dorsal corolla lobe. (**f**) Lateral view of corolla lobe. (**g**) Lateral staminodes. (**h**) Calyx and ovary. (**i**) Floral tube and stamen with calyx and ovary. (**j**) Back view of anther. (**k**) Front view of ovary. (**l**) Bract. (**m**) Bracteole. (**n**) Ovary with epigynous glands. (**o**) Labellum, flattened under a glass plate to show its fully expanded shape. Photographs (**a**,**b**) by Mukdamas Ruengrit and (**c**–**o**) by Thawatphong Boonma.

**Figure 3:**
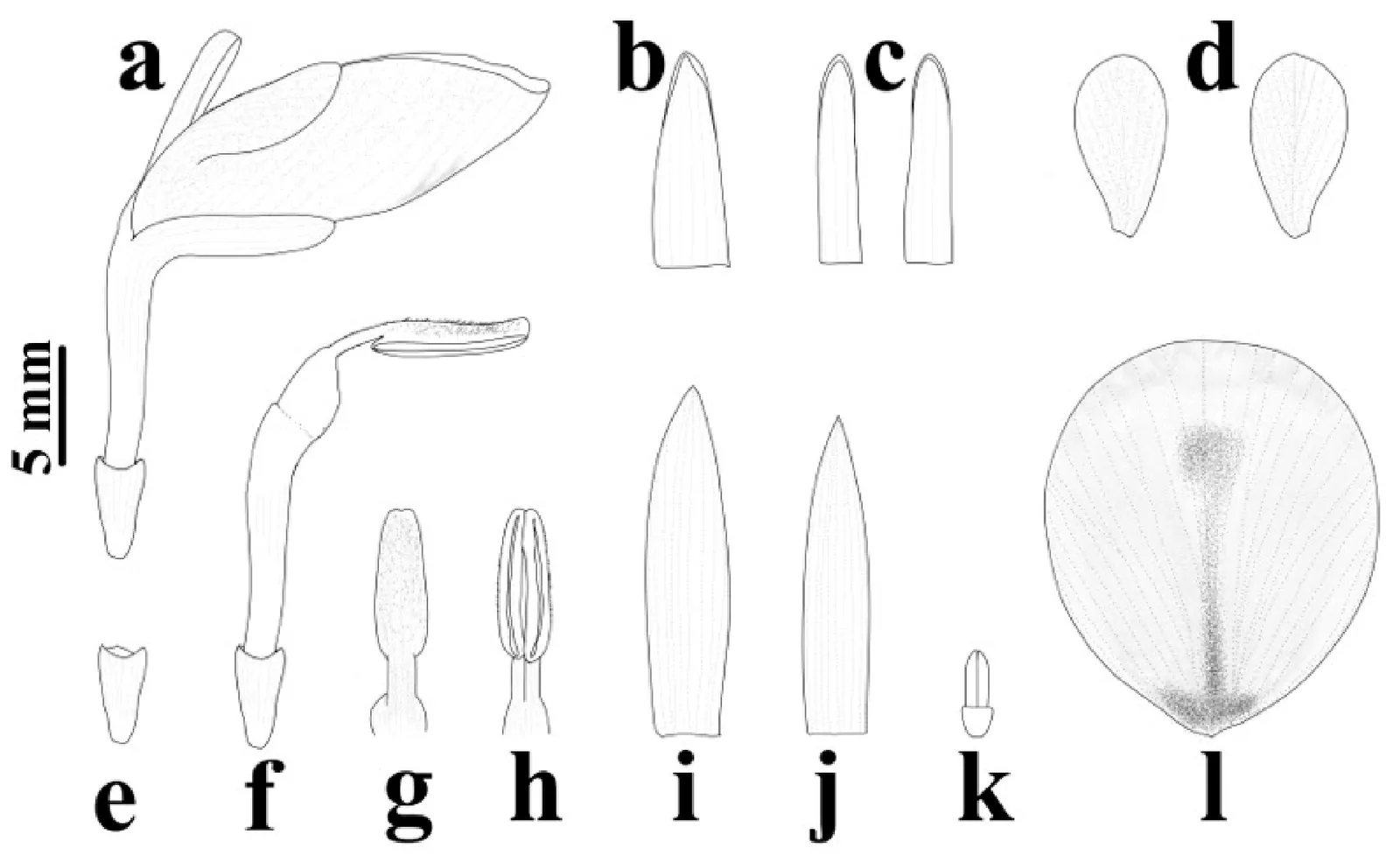
*Boesenbergia calcicola* Boonma, Saensouk & P.Saensouk, sp. nov. (**a**) Lateral view of flower. (**b**) Dorsal corolla lobe. (**c**) Lateral view of corolla lobe. (**d**) Lateral staminodes. (**e**) Calyx and ovary. (**f**) Floral tube and stamen with calyx and ovary. (**g**) Back view of anther. (**h**) Front view of ovary. (**i**) Bract. (**j**) Bracteole. (**k**) Ovary with epigynous glands. (**l**) Labellum, flattened to show its fully expanded shape. Drawn by Thawatphong Boonma.

**Figure 4:**
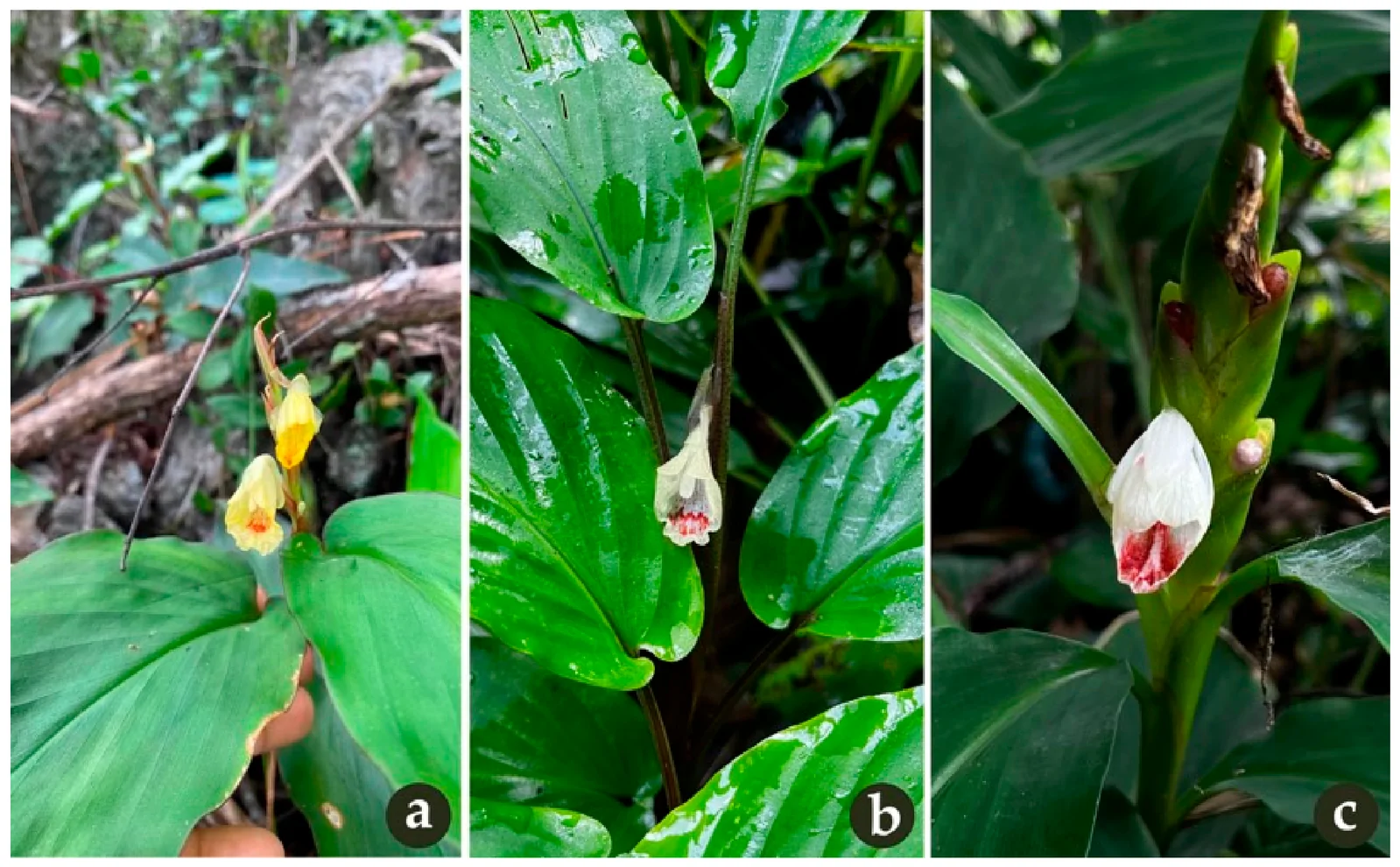
Comparison of habit and inflorescence morphology of *Boesenbergia calcicola* and morphologically similar species in their natural habitats: (**a**) *B. calcicola*; (**b**) *B. petiolata*; (**c**) *B. xiphostachya*. Photographs (**a**) by Mukdamas Ruengrit and (**b**,**c**) Thawatphong Boonma.

**Diagnosis.** *Boesenbergia calcicola* is morphologically similar to *B. petiolata* Sirirugsa in having a terminal inflorescence, lanceolate bracts, a broadly ovate to nearly orbicular labellum when flattened, and an anther crest absent, but differs by its inflorescence conspicuously exserted beyond the leafy shoot (vs. enclosed within or only shortly exserted from the upper leaf sheaths), shorter bracts, 1.5–2.5 cm long (vs. 3–4 cm long), shorter flowers, 2.0–2.5 cm long in lateral projection (vs. c. 4 cm long), a shorter calyx, 3.2–4.8 mm long (vs. c. 5 mm long), a floral tube 1.5–2.5 cm long and sparsely puberulent distally (vs. 2–3 cm long and glabrous), and smaller, puberulent lateral staminodes, 7.7–8.0 × 4.0–5.0 mm (vs. 12–13 × 8–9 mm and glabrous). The labellum of *B. calcicola* bears a conspicuous deep-red median band extending from the throat to approximately three-quarters of its length, broadening basally into a dark-maroon patch accompanied by a pair of dark-maroon triangular patches, whereas that of *B. petiolata* bears red markings extending along the veins toward the distal part of the labellum (Table 1, Figure 2, Figure 3 and Figure 4).

**Description:** Perennial rhizomatous herb, 35–40 cm tall. Primary rhizome ovoid, 1.1–1.5 × 0.8–1.2 cm, externally brown, internally ivory to cream. Roots fibrous, some bearing narrowly fusiform tubers tapering toward the rhizome. Stem 19–30 cm tall; bladeless sheath 2–3, 4–8 cm long, apex acute; leaf sheath distichous, red to vermilion basally, sometimes green distally, glabrous; ligule bilobed, 3.2–8.4 mm long, lobes triangular-ovate, apex acute to obtuse, vermilion or claret, glabrous; petiole 2.4–8.5 cm long, canaliculate, green or reddish tinged, glabrous. Leaves 3–5 per stem; lamina lanceolate, 6–15 × 3.0–5.5 cm, adaxially green, glabrous, minutely punctate, primary veins slightly prominent; abaxially pale green or with reddish tinged, glabrous, base cordate, apex acuminate, often minutely mucronulate, margin appearing entire to the naked eye but finely sinuate under a light microscope, hyaline, semitranslucent, pale green or red.

Inflorescence terminal, exserted beyond the leafy shoot, 9.0–13.0 cm long, peduncle 2.5–5 cm long, cylindrical, pale greenish white, glabrous, enclosed within the innermost leaf sheath; spike 6–8 cm long, with 7–15 flowers opening successively, usually one at a time. Bracts lanceolate, 1.5–2.5 × 0.4–0.8 cm, gradually decreasing in size toward the apex of the inflorescence, pale green to reddish brown tinged, glabrous, apex acute to acuminate. Bracteoles triangular-lanceolate, 1.2–2.4 × 0.35–0.7 cm, gradually decreasing in size toward the apex of the inflorescence, pale green, apex acute to acuminate, glabrous.

Flowers yellow, 2.0–2.5 cm long in lateral projection, measured in a straight line from the base of the ovary to the apex of dorsal corolla lobe. Calyx tubular–obconical 3.2–4.8 mm long, 2–3 mm in diameter distally, pale yellowish green, glabrous, apex equally trilobed, lobes 0.5–1.0 mm long, with angular apices formed by margins meeting at an angle greater than 90°, sinuses equal in depth. Floral tube cylindrical, curved at distal part, 1.5–2.5 cm long, yellow, sparsely puberulent at distal part; androecial cup c. 3 × 3 mm, orientated 90° to the floral tube, throat glabrous; dorsal corolla lobe lanceolate, 1.0–1.2 × 0.4–0.6 cm, yellow, glabrous, hooded, apex obtuse; lateral corolla lobes lanceolate, 0.9–1.1 × 0.35–0.5 cm, yellow, glabrous, hooded, apex obtuse. Lateral staminodes 2, obovate, 7.7–8.0 × 4.0–5.0 mm, yellow, puberulent, apex usually rounded, margin entire. Labellum broadly ovate to orbicular (flattened), slightly saccate, (1.7–)2.2–2.6 × (1.35–)2.0–2.3 cm (flattened), yellow, with a conspicuous longitudinal deep red median band extending from the throat to c. three-quarters of the labellum length and broadening basally into a dark maroon patch; throat with a pair of conspicuous dark maroon triangular patches flanking the basal part of the median band, apex rounded; margin entire, with slightly undulate apically. Stamen 1, 8.0–9.0 mm long; filament 1.8–2.0 × c. 1.5 mm, pale yellow, puberulent; anther 6.0–7.0 × 2.0–2.5 mm, yellow, puberulous; anther crest absent; anther thecae 6.0–6.9 mm long. Style filiform, 3.0–3.5 cm long, ivory; stigma elongate-obconical, ivory, ciliate. Epigynous glands 2, linear, 3–4 mm long, cream-colored. Ovary obovoid-oblate, 2.5–3.0 × c. 2.0 mm, pale green, glabrous, trilocular, with axile placentation. Fruit not seen.

**Vernacular name and utilization:** Locally known as “Krachai Khao Hin Pun”. The species is occasionally maintained by local residents as an ornamental pot plant, and limited local sale has recently been observed. No other uses were recorded during the present study. Although commercial availability currently appears limited, its ornamental appeal and emerging trade may increase collection pressure on the wild population and should therefore be considered in future conservation monitoring.

**Distribution and habitat:** *Boesenbergia calcicola* is currently known only from its type locality in Khon Kaen Province, Thailand. It grows in soil-filled crevices among limestone rocks in partially shaded karst forest at c. 385 m above sea level. Approximately 300 mature individuals were observed within the known locality. These plants were not concentrated in a single population patch but occurred discontinuously at several sites within the forest, generally forming small local aggregations of approximately 10–20 mature individuals. Plants occurring in the same or nearby habitat included *Boesenbergia collinsii* Mood & L. M. Prince, *Curcuma fimbriata* Škorničk. & Soonthornk, *Adiantum* spp., *Begonia* spp., and members of the Gesneriaceae family.

**Phenology:** Flowering observed in July in both 2025 and 2026; the flowering season is estimated to extend from June to September based on field observations of plants at different developmental stages. Fruit not seen.

**Etymology:** The specific epithet “*calcicola*” is derived from the Latin *calx* (genitive *calcis*), meaning “lime” or “limestone”, and *-cola*, meaning “dweller”, referring to the occurrence of the species on limestone substrates in Phu Pha Man District, Khon Kaen Province, northeastern Thailand.

**Additional specimen examined:** THAILAND, Northeastern, Khon Kaen Province, Phu Pha Man District, c. 385 m elevation, 31 July 2025, T. Boonma 2572, VMSU08900140 (VMSU); same locality, 13 July 2026, *T. Boonma* 2673, VMSU08900141 (preserved specimens) (VMSU).

### 3.2. Morphological Differentiation and Phenetic Similarity

Pairwise Gower distances showed that *Boesenbergia calcicola* was morphologically most similar to *B. xiphostachya* (0.27160), followed by *B. phengklaii* (0.28298) and *B. petiolata* (0.28692). Greater distances were obtained between *B. calcicola* and the remaining taxa, ranging from 0.35225 with *B. moodii* to 0.55288 with *B. sugudensis* (Table A3).

**Figure 5:**
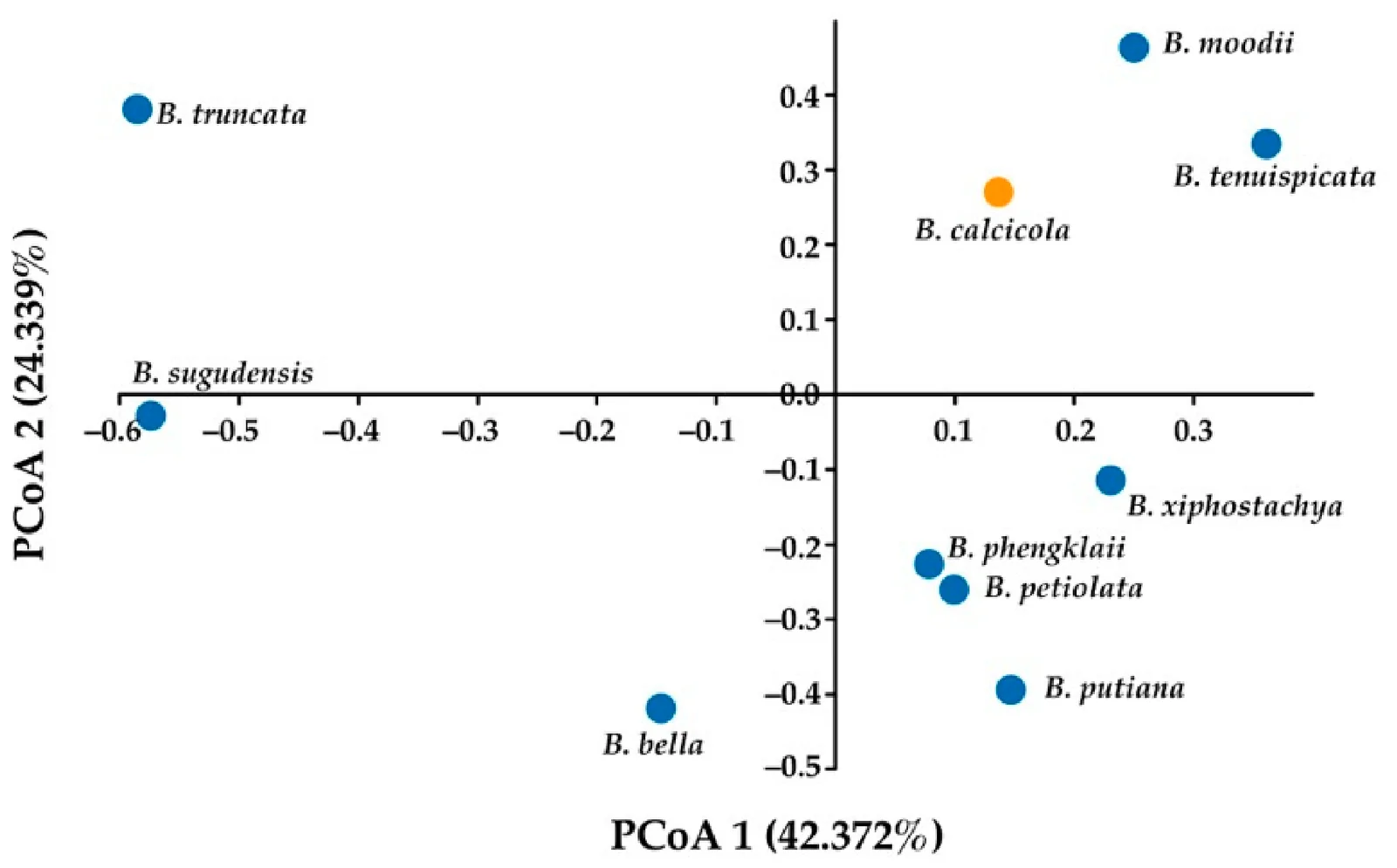
Principal coordinates analysis (PCoA) scatter plot based on Gower distance calculated from 32 selected morphological characters of *Boesenbergia calcicola* and selected morphologically similar taxa. The first two coordinate axes explain 66.711% of the total morphological variation, with PCoA 1 accounting for 42.372% and PCoA 2 for 24.339%.

Principal coordinates analysis (PCoA) based on the Gower distance matrix of 32 morphological characters showed that the first two axes explained 42.372% and 24.339% of the total variation, respectively, accounting for 66.711% in total (Figure 5). *Boesenbergia petiolata* and *B. phengklaii* remained positioned closest to one another in the lower-right quadrant, with *B. xiphostachya* and *B. putiana* also occurring on the positive side of PCoA axis 1. *Boesenbergia calcicola* occupied a distinct position in the upper-right quadrant, while *B. moodii* and *B. tenuispicata* were positioned farther upward and to the right in the same quadrant. *Boesenbergia truncata* and *B. sugudensis* were separated on the negative side of PCoA axis 1, whereas *B. bella* occupied the lower-left quadrant.

**Figure 6:**
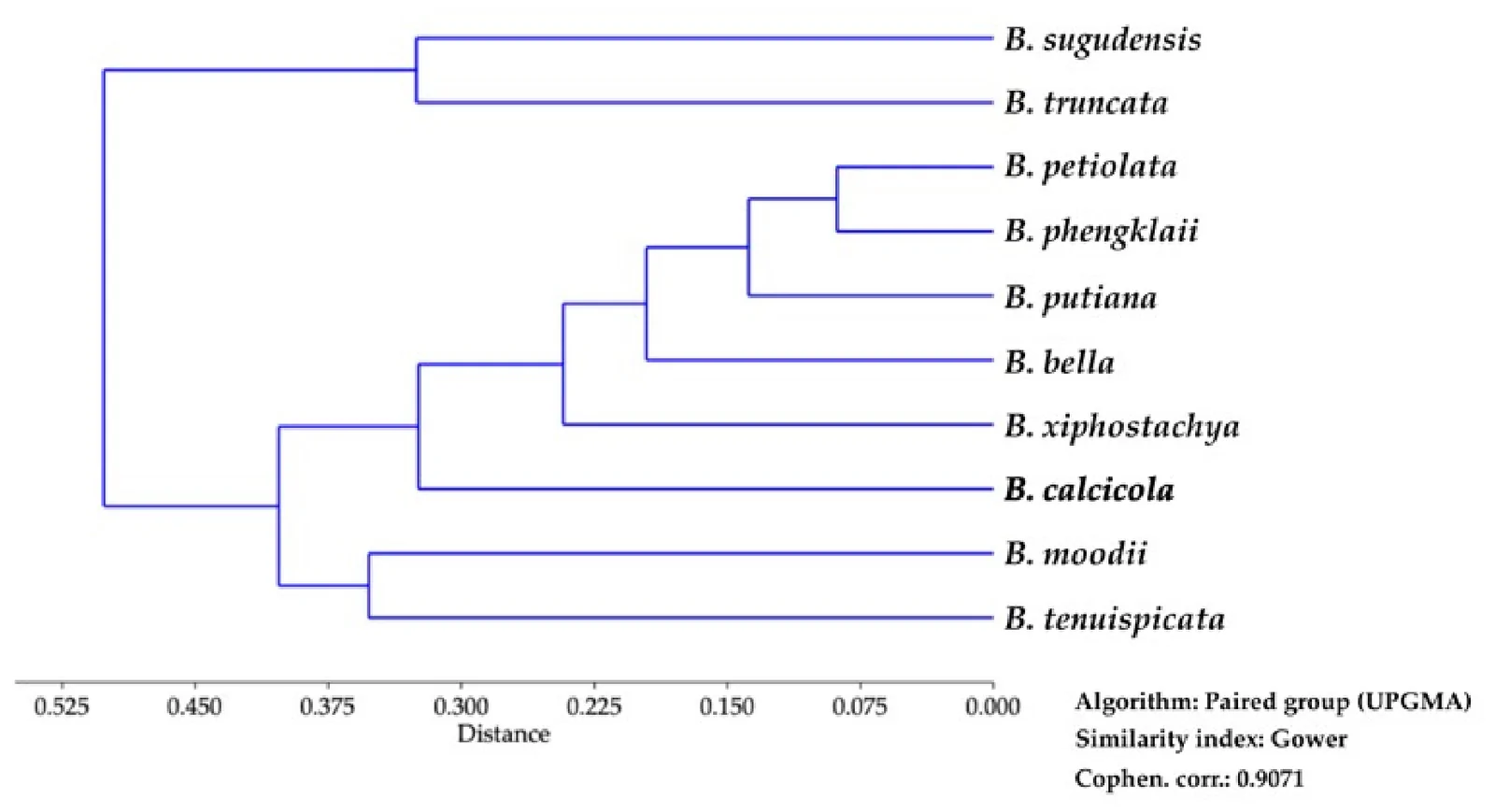
UPGMA dendrogram based on Gower distance calculated from 32 selected morphological characters of *Boesenbergia calcicola* and selected morphologically similar taxa. The cophenetic correlation coefficient is 0.9071. The dendrogram is interpreted as a phenetic summary of morphological similarity and not as a phylogenetic hypothesis.

The UPGMA dendrogram based on the Gower distance matrix of 32 morphological characters summarized the ten examined taxa into three principal phenetic clusters (Figure 6). Within the first cluster, *Boesenbergia petiolata* and *B. phengklaii* formed the closest pair, followed successively by *B. putiana*, *B. bella*, and *B. xiphostachya*. *Boesenbergia calcicola* subsequently joined this assemblage at a greater distance level, indicating that it remained morphologically differentiated from the more tightly grouped taxa within the cluster. The second cluster comprised *B. moodii* and *B. tenuispicata*, whereas *B. truncata* and *B. sugudensis* formed a third, more distinct cluster. The dendrogram had a cophenetic correlation coefficient of 0.9071, indicating a close correspondence between the UPGMA representation and the underlying Gower distance matrix.

### 3.3. Microhabitat and Preliminary Conservation Status

*Boesenbergia calcicola* is currently known only from the type locality in Phu Pha Man District, Khon Kaen Province, northeastern Thailand. The species occurs at approximately 385 m a.s.l. in a limestone-associated habitat, where plants grow in shallow soil-filled crevices among limestone rocks under partially shaded conditions. The occupied microsites are characterized by discontinuous soil interspersed with exposed limestone surfaces. 

Preliminary conservation status: Data Deficient (DD). The species is presently documented from a single locality, and suitable limestone habitats in the surrounding areas remain insufficiently surveyed. Consequently, its full geographical distribution, total population size, and population trend cannot yet be determined with confidence. Although the currently known distribution is highly restricted, this alone is insufficient for assignment to a threatened category under IUCN Criterion B without the additional conditions required by the criteria, such as continuing decline or extreme fluctuations. Therefore, *B. calcicola* is provisionally assessed as Data Deficient (DD) following the IUCN Red List Categories and Criteria [26]. Further surveys of suitable limestone habitats in Phu Pha Man District and adjacent areas are needed to clarify its distribution, population size, and potential threats.

## 4. Discussion

*Boesenbergia calcicola* is morphologically most comparable to *B. petiolata*, with which it shares a terminal inflorescence, lanceolate bracts, a broadly ovate to nearly orbicular labellum when flattened, and an anther crest absent [7,11,13,14]. Despite these similarities, the two species can be consistently distinguished by a combination of inflorescence and floral characters. *Boesenbergia calcicola* has an inflorescence conspicuously exserted beyond the leafy shoot, shorter bracts and flowers, a distally puberulent floral tube, smaller puberulent lateral staminodes, and a distinctive labellum color pattern. Its labellum is further characterized by a conspicuous deep-red median band extending from the throat to approximately three-quarters of its length, broadening basally into a dark-maroon patch accompanied by a pair of dark-maroon triangular patches. In *B. petiolata*, the inflorescence is enclosed within or only partly exposed from the upper leaf sheaths, the floral tube and lateral staminodes are glabrous, the lateral staminodes are larger, and the red labellar markings extend outward along the veins toward the distal portion. These differences are expressed across several floral and inflorescence structures rather than being restricted to variation in a single character (Table 1).

The distinction from *B. petiolata* is particularly important because morphological variation within that species has previously been documented. Reported variation includes plant robustness, lamina-base shape, reddish pigmentation of the abaxial leaf surface, the degree of yellow coloration in the flowers, the intensity of red coloration in the center of the labellum, and violet coloration near the labellum apex [11,12,15]. Accordingly, these variable vegetative and pigmentation characters were not treated as the principal basis for delimiting *B. calcicola*. Instead, the distinction is based primarily on the combination of inflorescence exsertion, floral dimensions, floral-tube and lateral-staminode indumentum, and labellar color pattern. This approach minimizes reliance on characters already documented as variable within *B. petiolata* and emphasizes a combination of morphological features that consistently distinguishes *B. calcicola* from that species.

The phenetic analyses summarize patterns of overall morphological similarity among the examined taxa and provide a quantitative representation of the same morphological dataset used in the taxonomic assessment. Based on the revised 32-character dataset, *B. calcicola* showed its lowest Gower distance with *B. xiphostachya* (0.27160), followed by *B. phengklaii* (0.28298) and *B. petiolata* (0.28692), with progressively greater distances from the remaining taxa. The relatively similar values among these three comparisons indicate that overall numerical similarity is distributed among several congeners rather than concentrated exclusively in a single species. This pattern does not conflict with the identification of *B. petiolata* as an important taxonomic comparison because the phenetic analysis summarizes overall similarity across all 32 scored characters, whereas taxonomic delimitation emphasizes combinations of diagnostically informative characters.

The PCoA and UPGMA analyses provide complementary representations of the same Gower distance dataset. The first two PCoA axes accounted for 66.711% of the total morphological variation, with PCoA 1 and PCoA 2 explaining 42.372% and 24.339%, respectively. *Boesenbergia calcicola* occupied a distinct position in the upper-right quadrant, separated in the ordination from the more closely positioned *B. petiolata*, *B. phengklaii*, *B. putiana*, and *B. xiphostachya*. Although *B. moodii* appeared relatively close to *B. calcicola* in the two-dimensional PCoA representation, its pairwise Gower distance from *B. calcicola* was 0.35225, whereas lower pairwise distances were recorded for *B. xiphostachya*, *B. phengklaii*, and *B. petiolata*. This apparent difference reflects the reduction in a multidimensional distance matrix to the first two coordinate axes; consequently, proximity in the two-dimensional ordination should not be interpreted as a direct ranking of pairwise morphological distances.

In the UPGMA analysis, *B. petiolata* and *B. phengklaii* formed the closest pair, followed successively by *B. putiana*, *B. bella*, and *B. xiphostachya*, with *B. calcicola* joining this assemblage at a greater distance level. The cophenetic correlation coefficient of 0.9071 indicates a close correspondence between the dendrogram and the underlying Gower distance matrix. Thus, the ordination and clustering analyses provide quantitative summaries of overall morphological similarity within the examined dataset. As defined in the analytical framework of this study, these analyses are not interpreted as independent evidence of species distinctness or as estimates of phylogenetic relationships, evolutionary independence, or reproductive isolation [16,17,24]. A limitation of the present study is the absence of molecular phylogenetic data. Future studies incorporating nuclear and plastid sequence data would provide an additional framework for evaluating the relationships of *B. calcicola* with morphologically similar congeners.

The occurrence of *B. calcicola* also contributes to the growing evidence that limestone karst landscapes in tropical Asia support highly localized plant diversity. Karst environments are characterized by pronounced fine-scale variation in substrate, exposed rock, soil development, topography, moisture availability, and shading, resulting in heterogeneous microhabitats that can support distinct plant assemblages [1,2,3]. Individual limestone outcrops may consequently harbor geographically restricted taxa, while the spatial fragmentation of these habitats can increase the vulnerability of localized populations to disturbance [1,4]. *Boesenbergia calcicola* is currently known from Phu Pha Man District, Khon Kaen Province, where it grows in soil-filled crevices among limestone rocks in a partially shaded habitat at approximately 385 m a.s.l. Its occurrence in this specialized habitat adds another limestone-associated member of *Boesenbergia* to the flora of northeastern Thailand. Future ecological studies could incorporate site-specific microclimatic measurements, physicochemical analyses of the substrate and soil, and assessments of associated microbial communities to better characterize the ecological requirements of *B. calcicola*.

The discovery is particularly noteworthy when considered together with the recent description of *B. lamudiae* from a localized limestone habitat in Loei Province [5]. Both discoveries resulted from the botanical exploration of limestone landscapes in northeastern Thailand and demonstrate that taxonomically distinctive *Boesenbergia* species can remain undocumented in localized karst habitats. This is consistent with continuing taxonomic discoveries and the documentation of *Boesenbergia* diversity across Southeast Asia [5,12,13,14,19]. Field examination of flowering plants is especially important in the genus because several diagnostically informative features involve floral configuration, color pattern, and fine indumentum, which may be difficult to evaluate completely from dried herbarium material [7,8,9,10]. In the present study, morphological observations and measurements were obtained directly from plants in the natural population and from spirit-preserved floral material collected from the same population, while colors were recorded from fresh material under natural light. This approach allowed diagnostically important floral characters of *B. calcicola* to be documented and compared directly with the available taxonomic evidence.

The currently known distribution of *B. calcicola* is restricted to the type locality, although limestone habitats in the surrounding region remain incompletely surveyed. Its known distribution should therefore be interpreted on the basis of verified records rather than assumed to represent its complete geographical range. The preliminary conservation assessment presented here follows the IUCN Red List framework and reflects the currently available information on distribution, population size, habitat specificity, and potential threats [26]. Additional botanical surveys of limestone habitats in Khon Kaen and adjacent areas, particularly during the observed flowering period from June to September, would help refine knowledge of its distribution and conservation status.

Taken together, the consistent combination of diagnostic morphological characters and the distinction from the documented range of variation in *B. petiolata* support the recognition of *B. calcicola* as a distinct species. The 32-character phenetic dataset provides a complementary quantitative summary of overall morphological similarity among the examined taxa. Its discovery further contributes to the documentation of localized *Boesenbergia* diversity associated with limestone habitats in northeastern Thailand and highlights the importance of continued flowering-season botanical exploration of these incompletely surveyed karst landscapes.

## 5. Conclusions

*Boesenbergia calcicola* is described as a new species from a limestone karst habitat in Phu Pha Man District, Khon Kaen Province, northeastern Thailand. Its recognition is supported by a consistent combination of morphological characters that distinguishes it from the morphologically similar *B. petiolata*, particularly the conspicuously exserted inflorescence, shorter bracts and flowers, distally puberulent floral tube, smaller puberulent lateral staminodes, and distinctive labellum color pattern. These differences remain distinct from the range of morphological variation previously documented in *B. petiolata*. Phenetic analyses based on 32 morphological characters provide a quantitative summary of overall morphological similarity among *B. calcicola* and the examined taxa, but are not treated as independent evidence for species delimitation. The species is currently known only from its type locality and is provisionally assessed as Data Deficient (DD) because its full distribution, population size, and population trend remain insufficiently known. The discovery of *B. calcicola*, together with other recently documented limestone-associated species of *Boesenbergia* in northeastern Thailand, highlights the importance of continued botanical exploration of limestone karst habitats for documenting geographically restricted plant diversity.

## Figures and Tables

**Table 1:** Diagnostic morphological comparison of *Boesenbergia calcicola*, *B. petiolata*, *B. phengklaii,* and *B. xiphostachya*.

Character	*B. calcicola*	*B. petiolata*	*B. phengklaii*	*B. xiphostachya*
Inflorescence	Terminal, conspicuously exserted beyond the leafy shoot	Terminal, basal portion clasped between the leaf sheaths, 1/4–1/2 exposed	Terminal, basal portion clasped between the leaf sheaths, 1/4–1/2 exposed	Terminal, exserted beyond the upper leaves
Peduncle length (cm)	2.5–5.0	c. 1	c. 1	c. 0.5
Spike length (cm)	6–8	c. 12	c. 9	12–17
Bract length (cm)	1.5–2.5	Up to 4	2.5–3.5	2–4
Bract color	Pale green or reddish-brown tinged	White to pale green with white	White with light green in the upper half	Dark green
Flower length (cm)	2.0–2.5	c. 4	c. 4.5	2.5–2.7
Calyx length (mm)	3.2–4.8	c. 5	c. 6	c. 6
Floral tube length (cm)	1.5–2.5	2–2.8	c. 3.8	c. 1.5
Floral tube indumentum	Sparsely puberulent distally	Glabrous	Glabrous	Glabrous
Lateral staminode size (mm)	7.7–8.0 × 4.0–5.0	c. 13 × 9	c. 15 × 10	c. 12 × 5
Lateral staminode color	Yellow	White	White	White
Lateral staminode indumentum	Puberulent	Glabrous	Internally glabrous; externally with short glandular hairs	Glabrous
Labellum shape	Broadly ovate to orbicular when flattened, slightly saccate	Nearly orbicular when flattened, deeply saccate	Broadly elliptical when flattened, deeply saccate	Broadly obovate, saccate
Labellum size (cm)	(1.7–)2.2–2.6 × (1.35–)2.0–2.3	2.5–2.8 × 2.0–2.5	c. 2.8 × 2.5	c. 3 × 2
Labellum ground color	Yellow	White, margins becoming yellowish with age	White, becoming yellowish with age, especially along the margins	White
Labellum indumentum	Puberulent	Externally with a few short glandular hairs	Externally covered with short glandular hairs	Glabrous
Labellum markings	Conspicuous deep-red median band extending from the throat to c. 3/4 of the labellum length; basal dark-maroon patch and paired dark-maroon triangular throat patches	Two red throat spots, with red spots and longitudinal markings extending outward along the veins toward the distal portion	Throat marked with red; maculate red pattern extending outward on either side of the center to c. 3/4 of the labellum length, forming a broad rectangular area; center white with a red margin, followed distally by red stripes interspersed with violet	Orange-red throat, the coloration broadening toward the apex
Extent of principal red marking	Terminates distinctly before the apex, at c. 3/4 of labellum length	Red markings extend toward the distal/apical portion	Maculate red pattern extends to c. 3/4 of the labellum length; red stripes continue toward the apex, interspersed with violet	Extends from the throat and broadens toward the apex
Androecial cup	Present, c. 3 × 3 mm	Present, c. 2 × 2 mm	Present, c. 2 × 2 mm	Present, c. 4 mm long
References	Present study	[7,11]	[15]	[7,14]

Note: Morphological data for *B. calcicola* were obtained from living plants and preserved material examined in the present study. Data for *B. xiphostachya* were compiled from the treatment in the Flora of Thailand [7] and the recent taxonomic account based on Cambodian material [14]. Data for *B. petiolata* primarily follow the most recent treatment in the Flora of Thailand [7], with the original taxonomic account of Sirirugsa [11] additionally consulted. Data for *B. phengklaii* were derived from its original taxonomic description and verified against available type information [15].

## Data Availability

The morphological character definitions and coding scheme, coded character-state matrix, and Gower distance matrix used in the phenetic analyses are provided in Table A1, Table A2 and Table A3. Additional data supporting the findings of this study are available from the corresponding authors upon reasonable request.

## Abbreviations

The following abbreviations are used in this manuscript:

A	Harvard University, Cambridge, Massachusetts, USA
AAU	Aarhus University, Denmark
B	Botanic Garden and Botanical Museum Berlin-Dahlem, Germany
BK	Bangkok Herbarium, Department of Agriculture, Thailand
BKF	Department of National Parks, Wildlife and Plant Conservation, Thailand
BM	The Natural History Museum, London, England, UK
BORH	Universiti Malaysia Sabah, Kota Kinabalu, Sabah, Malaysia
C	University of Copenhagen, Copenhagen, Denmark
CAL	Botanical Survey of India, Howrah, West Bengal, India
DMSC	Medicinal Plants Research Institute, Thailand
E	Royal Botanic Garden Edinburgh, Edinburgh, UK
HITBC	Xishuangbanna Tropical Botanical Garden, Academia Sinica, Yunnan, China
HN	Vietnam Academy of Science and Technology (VAST), Hanoi, Vietnam
HNPM	National Institute of Medicinal Materials, Vietnam
HNU	VNU University of Science, Hanoi, Vietnam
IBK	Guangxi Institute of Botany, China
IMDY	Chinese Academy of Medical Sciences, China
K	Royal Botanic Gardens, UK
KKU	Khon Kaen University Herbarium, Thailand
L	Naturalis Biodiversity Center, Leiden, The Netherlands
NY	The New York Botanical Garden, New York, USA
P	Muséum National d’Histoire Naturelle, Paris, France
QBG	Queen Sirikit Botanic Garden, The Botanical Garden Organization, Thailand
RAF	Forest Research Institute, Yezin, Nay Pyi Taw, Myanmar
SGN	Southern Institute of Ecology, Ho Chi Minh City, Vietnam
SING	Singapore Botanic Gardens, National Parks Board, Singapore
TNS	National Museum of Nature and Science, Tokyo, Japan
US	Smithsonian Institution, Washington, Columbia, USA
VMSU	Vascular Plant Herbarium, Mahasarakham University, Thailand
VNM	Institute of Life Science, Ho Chi Minh City, Vietnam
VNMN	Vietnam National Museum of Nature, Hanoi, Vietnam

## Appendix A. Morphological Data Used in the Phenetic Analyses

The appendix provides detailed information supporting the phenetic analyses presented in this study, including the definition and coding of morphological characters, the morphological character matrix, and the Gower distance matrix. These materials are provided to facilitate transparency and reproducibility of the phenetic analyses.

**Table A1:** Morphological characters and coding scheme used for the phenetic analyses.

Code ^1^	Category	Character	Data Type ^2^	States/Coding Rule
UG1	UG	Rhizome architecture	Nominal	0 compact/ovoid; 1 elongate; 2 branched; 3 linearly expanding; 4 clustered elements; 5 bud-crowned tuberous roots
UG2	UG	Tuberous roots	Binary	0 absent; 1 present
UG3	UG	Tuber form	Nominal	0 absent; 1 fusiform; 2 cylindrical/elongate; 3 terminally spheroid; 4 variable/mixed
VG1	VG	Ligule form	Nominal	0 entire; 1 bilobed; 2 auriculate-bilobed; 3 caudate
VG2	VG	Maximum lamina width (cm)	-	Maximum reported value
IN1	IN	Inflorescence position	Nominal	0 radical; 1 terminal; 2 radical initially, later terminal
IN2	IN	Degree of inflorescence exsertion	Ordinal	0 enclosed/slightly exposed; 1 partly exposed; 2 mostly exserted; 3 prominently exserted
IN3	IN	Maximum peduncle length (cm)	-	Maximum reported value
IN4	IN	Maximum spike length (cm)	-	Maximum reported value
IN5	IN	Maximum bract length (cm)	-	Maximum reported value
IN6	IN	White ground color in bracts	Binary	0 absent; 1 present
IN7	IN	Bract indumentum	Binary	0 glabrous; 1 pubescent
IN8	IN	Maximum bracteole length (cm)	-	Maximum reported value
IN9	IN	Bracteole indumentum	Binary	0 glabrous; 1 pubescent
FL1	FL	Maximum flower length (cm)	-	Maximum reported value
FL2	FL	Flower ground color	Nominal	0 white; 1 yellow; 2 orange-yellow; 3 variable pale white/yellow/greenish
FL3	FL	Maximum calyx length (mm)	-	Maximum reported value
FL4	FL	Calyx apex	Nominal	0 entire/irregularly entire; 1 bifid/bilobed; 2 trifid/trilobed; 3 rounded
FL5	FL	Calyx indumentum	Binary	0 glabrous; 1 pubescent
FL6	FL	Maximum floral-tube length (cm)	-	Maximum reported value
FL7	FL	Floral-tube indumentum	Binary	0 glabrous; 1 pubescent at least distally
FL8	FL	Maximum labellum length (cm)	-	Maximum reported value
FL9	FL	Maximum labellum width (cm)	-	Maximum reported value
FL10	FL	Labellum ground color	Nominal	0 white; 1 yellow; 2 orange-yellow; 3 variable pale/greenish yellow
FL11	FL	Labellum color-pattern type	Nominal	0 absent/plain; 1 localized throat patch/spots; 2 dispersed maculate/striped/banded; 3 continuous longitudinal median band; 4 median band plus paired basal blotches
FL12	FL	Maximum lateral-staminode length (cm)	-	Maximum reported value
FL13	FL	Maximum lateral-staminode width (cm)	-	Maximum reported value
FL14	FL	Lateral-staminode ground color	Nominal	0 white; 1 yellow; 2 orange-yellow; 3 variable pale white/yellow
FL15	FL	Lateral-staminode indumentum	Binary	0 glabrous; 1 pubescent/puberulent
FL16	FL	Maximum anther length (mm)	-	Maximum reported value
FL17	FL	Anther indumentum	Binary	0 glabrous; 1 hairy/glandular hairy
FL18	FL	Anther crest	Binary	0 absent; 1 present

^1^ Character codes indicate the organ category from which each character was derived: UG = underground organs, VG = vegetative characters, IN = inflorescence characters, and FL = floral characters. The category column repeats these groupings for ease of reference. ^2^ Data types follow the PAST coding convention: “–” = continuous numerical variable; “Ordinal” = ordered categorical variable; “Nominal” = unordered categorical variable; and “Binary” = two-state variable coded as 0/1.

**Table A2:** Morphological character matrix of the *Boesenbergia* taxa included in the phenetic analyses.

Taxon	UG1	UG2	UG3	VG1	VG2	IN1	IN2	IN3	IN4	IN5	IN6	IN7	IN8	IN9	FL1	FL2	FL3	FL4	FL5	FL6	FL7	FL8	FL9	FL10	FL11	FL12	FL13	FL14	FL15	FL16	FL17	FL18
*B. bella*	2	1	2	2	14	1	1	0.5	11	4.5	1	1	4.7	0	4.5	0	8	1	1	2.5	0	2.2	2	0	2	1	0.5	0	0	8	1	0
*B. calcicola*	0	1	1	1	5.5	1	3	5	8	2.5	0	0	2.4	0	2.5	1	4.8	2	0	2.5	1	2.6	2.3	1	4	0.8	0.5	1	1	7	1	0
*B. moodii*	1	0	0	1	7	1	3	5	15	8	0	0	2.5	0	5	2	10	3	0	3	0	5	3.5	2	1	1.7	1.5	2	1	6	0	0
*B. petiolata*	3	1	2	1	8	1	1	1	12	4	1	0	4	0	4	0	5	2	0	2.8	0	2.8	2.5	0	2	1.3	0.9	0	0	8	1	0
*B. phengklaii*	3	1	3	1	8	1	1	1	9	3.5	1	0	3.2	0	4.5	0	6	1	0	3.8	0	2.8	2.5	0	2	1.5	1	0	1	8	1	0
*B. putiana*	3	1	4	1	14	1	1	1	13	6	1	0	6	0	5	0	10	1	0	3	0	3.2	2.6	0	2	2	1.1	0	1	8	1	0
*B. sugudensis*	1	0	0	3	7.5	1	1	0.8	7.2	6.5	1	1	3.8	1	5.7	0	10	1	1	4.6	1	1.3	1	0	2	1.5	0.5	0	0	3	0	1
*B. tenuispicata*	4	0	0	1	10	2	2	15	13	3	0	0	1.5	0	5	3	4	0	0	3	0	2.2	1.7	3	1	1.3	1.1	3	0	7	1	0
*B. truncata*	1	0	0	0	3.6	1	1	0.45	2.5	3	1	1	2	1	2.3	0	4	0	1	0.8	1	0.6	0.5	1	0	0.3	0.1	0	1	2	0	0
*B. xiphostachya*	5	1	4	1	8	1	3	0.5	17	4	0	0	3	0	2.7	0	6	0	0	1.5	0	3	2	0	1	1.2	0.5	0	0	7	1	0

**Table A3:** Pairwise Gower distance matrix among the ten *Boesenbergia* taxa calculated from 32 morphological characters.

Species	*B. calcicola*	*B. xiphostachya*	*B. phengklaii*	*B. petiolata*	*B. moodii*	*B. putiana*	*B. bella*	*B. tenuispicata*	*B. truncata*	*B. sugudensis*
*B. calcicola*	0.00000	0.27160	0.28298	0.28692	0.35225	0.38524	0.39443	0.39801	0.44620	0.55288
*B. xiphostachya*	0.27160	0.00000	0.21881	0.18852	0.40779	0.27969	0.28338	0.33918	0.52739	0.54233
*B. phengklaii*	0.28298	0.21881	0.00000	0.08828	0.39034	0.11542	0.20867	0.38694	0.47506	0.43572
*B. petiolata*	0.28692	0.18852	0.08828	0.00000	0.41039	0.16082	0.16514	0.35323	0.49984	0.42739
*B. moodii*	0.35225	0.40779	0.39034	0.41039	0.00000	0.37652	0.51691	0.35216	0.58235	0.52040
*B. putiana*	0.38524	0.27969	0.11542	0.16082	0.37652	0.00000	0.21323	0.44629	0.57733	0.46354
*B. bella*	0.39443	0.28338	0.20867	0.16514	0.51691	0.21323	0.00000	0.45739	0.44743	0.32528
*B. tenuispicata*	0.39801	0.33918	0.38694	0.35323	0.35216	0.44629	0.45739	0.00000	0.59604	0.60598
*B. truncata*	0.44620	0.52739	0.47506	0.49984	0.58235	0.57733	0.44743	0.59604	0.00000	0.32532
*B. sugudensis*	0.55288	0.54233	0.43572	0.42739	0.52040	0.46354	0.32528	0.60598	0.32532	0.00000

**Table A4:** Specimens and type materials examined or consulted in the taxonomic assessment of *Boesenbergia calcicola*.

Taxon	Specimen Status	Collector & Collection no.	Herbarium	Locality	Remarks
*B. calcicola*	Holotype	T. Boonma 2571	VMSU	Thailand, Northeastern, Khon Kaen, Phu Pha Man	Present study (VMSU 00000014)
*B. calcicola*	Examined specimen	T. Boonma 2572	VMSU	Thailand, Northeastern, Khon Kaen, Phu Pha Man	Present study (VMSU 08900140)
*B. calcicola*	Examined specimen	T. Boonma 2673	VMSU	Thailand, Northeastern, Khon Kaen, Phu Pha Man	Present study (preserved material; VMSU 08900141)
*B. xiphostachya*	Lectotype	Pierre s.n.	P	Laos, Ri–Hao	Collected in September 1865; lectotype of *Gastrochilus xiphostachyus* Gagnep., designated by Saensouk et al. [14]
*B. xiphostachya*	Examined specimen	T. Boonma BC001	VMSU	Cambodia, Preah Vihear, near the road to Preah Vihear Mt.	Specimen cited in Saensouk et al. [14], incorporated into the comparative morphological assessment
*B. xiphostachya*	Examined specimen	D. Song BC005	VMSU	Cambodia, Preah Vihear, Phnom Tbeng Meanchey	Specimen cited in Saensouk et al. [14], incorporated into the comparative morphological assessment
*B. phengklaii*	Holotype	J.D. Mood 16P08	BKF	Thailand, Northern, Chiang Mai, Doi Suthep foothills	Originally collected in the field as Mood & Vatcharakorn 3349; cultivated in Hawaii, USA as M3349; voucher prepared on 1 October 2016 as Mood 16P08 and selected as the type [15]
*B. petiolata*	Holotype	Maxwell 74–631	BK	Thailand, Central, Saraburi, Sahm Lahn, rocky bamboo-hardwood forest	Sirirugsa [11]
*B. moodii*	Holotype	Chit Soe Paing 001	RAF	Myanmar, Rakhine State, around Dal Poke Village, Kyay Chaing village tract, Gwa Township, Thandwe District	Tanaka et al. [13]
*B. putiana*	Holotype	J.D. Mood 16P09	BKF	Thailand, Northern, Mae Hong Son, Mueang, near Tam Pla Pha Suea cave	Originally collected in the field as Mood & Vatcharakorn 3360; cultivated in Hawaii, USA as M3360; voucher prepared on 1 October 2016 as Mood 16P09 and selected as the type [15]
*B. bella*	Holotype	J.D. Mood 16P07	BKF	Lao P.D.R., North of Pakse	Originally collected in Lao P.D.R. as Paprataro s.n.; subsequently cultivated in Hawaii, USA as J.D. Mood 3397; voucher prepared on 1 October 2016 as J.D. Mood 16P07 and selected as the type [15]
*B. tenuispicata*	Holotype	Larsen et al. 43446	AAU	Thailand, Peninsular, Krabi, Than Bok Koroni at Ao Luk	Larsen [27]
*B. truncata*	Holotype	Lam Nyee Fan 342	BORH	Malaysia, Borneo, Sabah, cultivated at Kipandi Park, Moyog	Vouchered on 12 October 2016 from cultivated material. Original wild material was collected by Linus Gokusing BS-09, c. 100 m west of Kipandi Park, Sabah [20]
*B. sugudensis*	Holotype	Lam Nyee Fan 356	BORH	Malaysia, Borneo, Sabah, cultivated at Kipandi Park, Moyog	Vouchered on 12 October 2016 from cultivated material. Original wild material was collected by Linus Gokusing BS-23 at Kampung Sugud, Penampang, Sabah [20]
